# Sodium bicarbonate and beta-alanine supplementation: Is combining both better than either alone? A systematic review and meta-analysis

**DOI:** 10.5114/biolsport.2024.132997

**Published:** 2024-01-02

**Authors:** Terence Curran-Bowen, André Guedes da Silva, Gabriel Barreto, John Buckley, Bryan Saunders

**Affiliations:** 1 Centre for Active Living, University Centre Shrewsbury, University of Chester, Shrewsbury, UK; 2 Applied Physiology and Nutrition Research Group, School of Physical Education and Sport; Rheumatology Division; Faculdade de Medicina FMUSP, Universidade de São Paulo, São Paulo, SP, BR, University of São Paulo, SP, Brazil; 3 Keele University, School of Allied Health Professions, Newcastle-under-Lyme, UK; 4 Institute of Orthopaedics and Traumatology, Faculty of Medicine FMUSP, University of São Paulo, Brazil; 5 Nutrology Academy, Rio de Janeiro, Brazil

**Keywords:** Ergogenic Aids, Fatigue, Nutrition, Sport, Exercise, Performance

## Abstract

This systematic review and meta-analysis aimed to determine the effect of combined beta-alanine (BA) and sodium bicarbonate (SB) supplementation on exercise capacity and performance. Four databases (PubMed, SPORTDiscus, Web Of Science and MEDLINE) were searched using relevant terms for studies involving healthy (e.g. no chronic diseases or conditions) male or female adults of any training status (athletes, physically active and non-athletes) and that investigated BA and SB in isolation and combination at any dose on an exercise outcome. Ten studies, totalling 243 individuals, met the criteria with 12 outcomes for each nutritional supplement. No ergogenic effect was detected in this meta-analysis for BA (SMD = 0.18, 95% CI: -0.06; 0.43, p = 0.13, tau^2^ = 0, tau = 0, I^2^ = 0.0%) or SB (SMD = 0.17, 95% CI: -0.08; 0.41, p = 0.16, tau^2^ = 0, tau = 0, I^2^ = 0.0%) in isolation. However, there was a beneficial effect for the combination of BA and SB (SMD = 0.32, 95% CI: 0.07; 0.57, p = 0.02, tau^2^ = 0, tau = 0, I^2^ = 0.0%). Meta-regression identified no differences between supplementing with BA or SB separately (F = 0.58; p = 0.57). Combining BA and SB improved exercise performance, however, there was no benefit in taking these supplements individually.

## INTRODUCTION

High-intensity exercise relies on substantial energy supply from an-aerobic metabolism [1], with a concomitant increase in hydrogen ions (H^+^) due, at least in part, to imbalance in the strong ion difference [2]. Accumulation of H^+^ can negatively impact exercise performance due to the reduction in muscle pH, namely muscle acidosis. A low muscle pH has been shown to affect muscle contractability by inhibiting glycolytic enzymes [3], increasing competition with calcium ions from the sarcoplasmic reticulum [4] and interfering with actin and myosin cross-bridge formation [5]. These lead to the muscle being unable to generate or maintain force which results in fatigue. The body has several physiological mechanisms, namely intracellular and extracellular buffering systems [6], that work to best maintain pH homeostasis during exercise, and these can be manipulated nutritionally to enhance their relative contributions.

Beta-alanine combines with L-histidine to create the dipeptide carnosine in muscle, which acts as an intracellular buffer against H^+^ accumulation [7]. Supplementation of beta-alanine at doses of 3.2 to 6.4 g/day for 4–12 weeks has consistently been shown to increase muscle carnosine content, with increases expected in all individuals [8]. Thus, beta-alanine supplementation is an appropriate method to enhance the intracellular buffering capacity of our muscles. The bicarbonate buffering system is the most important system for maintaining pH homeostasis in the blood. Sodium bicarbonate supplementation can increase the concentration of circulating bicarbonate [9], with 0.3 g/kg body mass (BM) the most commonly employed dose in the literature [10]. Increased circulating bicarbonate increases the pH of the blood and also increases the transport of H^+^ out of the exercising muscle [11], thereby indirectly contributing to the buffering capacity of the muscle. Enhancing these buffering systems via supplementation can support an athlete to maintain an elevated level of exercise performance.

Sodium bicarbonate and beta-alanine are both considered to be effective ergogenic supplements, a growing volume of reviews and meta-analytic evidence supporting their roles in improving athletic performance and capacity [10; 12; 13; 14; 15; 16; 17; 18; 19]. Improvements with beta-alanine or sodium bicarbonate have been shown in various exercise modalities, including rowing [20; 21; 22; 23], swimming [16; 24; 25], cycling [26; 27; 28; 29], running [30; 31], team sports [32; 33] and combat sports [34; 35; 36]. These intracellular and extracellular buffering systems work simultaneously to maintain the homeostatic pH of the muscle, meaning that co-ingestion of beta-alanine and sodium bicarbonate may lead to additional improvements in exercise performance compared to taking either supplement in isolation. However, evidence for additional effects is unclear, with some studies showing strong additive effects of co-supplementation [37], others showing potential further benefits [38; 39], while others showed no additional benefit when beta-alanine and sodium bicarbonate were combined [40; 41]. A meta-analysis showed that adding sodium bicarbonate to beta-alanine led to greater performance gains than beta-alanine alone [14], but no comparison was made to sodium bicarbonate alone, hindering conclusions. Since recreational and trained athletes will likely consume a combination of different supplements in order to maximise performance [42], further exploration of the combined effects of ergogenic aids would provide a more thorough and practical understanding of how these substances interact to affect performance.

To date, there has been no systematic review and meta-analysis investigating the effect of the co-ingestion of beta-alanine or sodium bicarbonate. Therefore, the aim of this paper was to perform a systematic review of the current evidence and meta-analyse the data to determine if there is an additive benefit of co-supplementation of beta-alanine and sodium bicarbonate on exercise performance and capacity above either supplement alone.

## MATERIALS AND METHODS

### Study Eligibility

This systematic review with meta-analyses followed the PRISMA-P statement [43] protocols and the research framed using PICOS (Population, Intervention, Comparator, Outcomes, Study Design) [44]. The PRISMA checklist can be found as a Supplementary File. Only peerreviewed experimental studies were included; unpublished documents, review studies, conference abstracts, theses, and book chapters were excluded. Study inclusion criteria were based on the PICOS framework, (i) population, (ii) intervention, (iii) comparisons, (iv) outcomes, and (v) study design. Inclusion criteria were as follows: i) studies involving healthy adults only (*e.g.*, no chronic diseases or conditions) of both sexes and any training status (athletes, physically active and nonathletes); ii) intervention session with sodium bicarbonate and betaalanine in both isolation and combination at any dose; iii) comparison to a placebo; iv) exercise capacity or performance-related outcome(s); v) study trials with any experimental design. The exclusion criteria comprised: i) studies that recruited participants with any illness or physical restriction; ii) studies that did not measure exercise capacity or performance; iii) studies with blended supplements including substances other than beta-alanine and sodium bicarbonate.

### Search Strategy

An online search of the literature was conducted in May 2021 and repeated again in February 2023 using the following databases: PubMed, SPORTDiscus, Web Of Science and MEDLINE. The Boolean operators and key words were, “(beta alanine OR beta-alanine OR β-alanine) AND (sodium bicarbonate) AND (exercise OR sport OR performance)”. No year restriction was adopted. Search results were uploaded to an online platform (Rayyan software, Qatar Computing Research Institute, Qatar) [45] to perform the screening process.

**FIG. 1:**
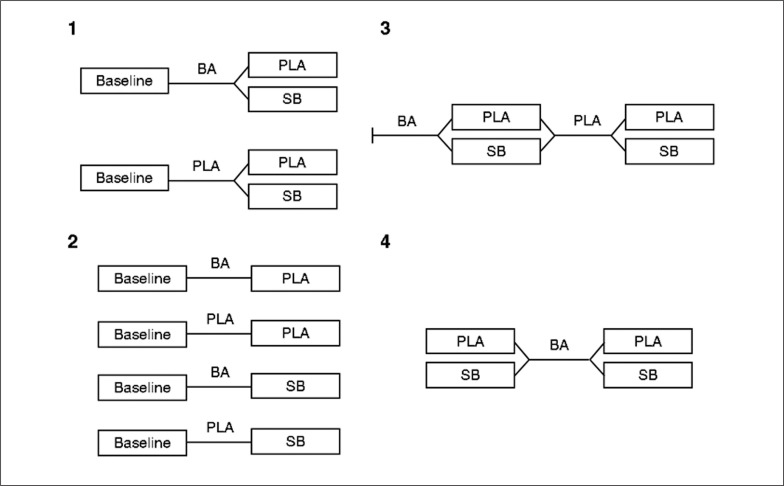
Visual representation of each of the 4 study designs encountered. BA = Beta-alanine, SB = Sodium bicarbonate, PLA = Placebo.

Following the removal of duplicates, two reviewers (TCB and AGS) independently assessed the eligibility of the title and abstract of all the results provided by the search based on the inclusion and exclusion criteria. The separate selection of papers was then compared, with no conflicts. The next stage consisted of obtaining and evaluating the entire remaining articles against the inclusion/exclusion criteria. Where there were any conflicts about suitability, a decision was made following discussion with a third reviewer (BS). The search and screening strategy is depicted in Figure 1.

### Data Extraction

The total number of articles were randomly halved and assigned to two authors (TCB and AGS) who each extracted data from their assigned studies, before each cross-checking the other’s extracted data to avoid any errors. The following data was extracted from each selected studies: author and year of publication, population characteristics (sex, age, training status), experimental design, supplementation protocols (dose, timing), exercise protocol and exercise related outcome data. All the extracted data was organized in standardized Microsoft Excel spreadsheets. A single exercise outcome measure was extracted from each exercise protocol to ensure consistency in data analysis and avoid data repetition based on a hierarchy of a previous meta-analysis with beta-alanine supplementation and exercise [14]. The outcome data hierarchy was as follows: (i) total work; (ii) mean output throughout the test (i.e., mean power output; mean velocity; mean jump height); and (iii) time to completion (performance test)/time to exhaustion (capacity test). When data was available in the form of a figure, means and standard deviations were extracted with the *digitize* package in the Rstudio Software (Rstudio 1.4.1103, PBC, USA).

Four types of study designs were identified and are demonstrated in Figure 1. The four types included the following: 1) two parallel groups for beta-alanine with 1 baseline measure and a crossover between a placebo and a sodium bicarbonate condition; 2) four parallel groups (PLA/PLA, BA/PLA, PLA/SB, BA/SB) with individual baseline measures; 3) a crossover (PLA/BA) without baseline measures, but with a final crossover trial between PLA and SB and 4) a single group receiving beta-alanine with baseline and post trials with both PLA and SB.

### Analysis

Obtained means and standard deviations (SDs) were transformed into standardized mean differences (SMDs, Hedge’s *g*). The way in which the SMDs were calculated varied according to the study design. For pre-post design studies (designs 1 and 2) [1; 3; 4; 6; 7; 8; 9; 10], SMDs were calculated as per the d_ppc2_ effect size and variance σ^2^(_dppc2_) described in Morris [46]. Since we did not have access to full data-sets, and therefore could not calculate the correlation component of the variance estimate σ^2^(dppc2), the suggested value of 0.7 was utilized. For pooled SD calculations, only baseline SD values were inputted.

Whenever studies utilized a cross-over model (study designs 3 and 4), SMDs and standard errors (SEs) were calculated utilizing simple pairwise group comparisons between means and standard deviations from experimental vs. placebo conditions (*esc_mean_sd* function from the *esc* package). A correlation of 0.7 was also inputted for these comparisons. Pooled SDs were calculated utilizing only the SDs of placebo conditions, since variances are expected to always be homogenous. In cases which a repeated measures design was utilized [25], SMDs were calculated individually for each measure and then combined as described by [47]. A correlation of 0.7 was also used for the combination calculus.

A three-level meta-analysis was then performed (*metagen* function from the *meta* package) with all the SMDs derived from separate studies with the restricted maximum-likelihood estimator (REML) for the obtention of variance estimates tau and tau-squared. This model was chosen due to its capacity to consider a within-study variance component, which accounts for the existence of more than one exercise outcome per study, which was the case, since comparisons were made between outcomes of single studies (BA/PLA vs. BA/SB, etc.). Random effects estimates were adjusted with the Hartung-Knapp method [48]. Confidence intervals for these estimates were calculated with the Profile-likelihood method for three-level meta-analyses [49]. A meta-regression was performed with supplement type as fixed factor with three levels (BA, SB or BA+SB) to investigate whether there are additional effects of supplementing BA and SB together and to compare the effects of both supplements separately.

Potential bias was assessed through a subjective evaluation of a funnel plot and, if any asymmetry was evident, specific tests were performed (Egger’s test for small-study bias). All analyses were performed with the Rstudio Software (Rstudio 1.4.1103, PBC, USA). Statistical significance was previously set at p < 0.05.

## RESULTS

### Search strategy

**FIG. 2:**
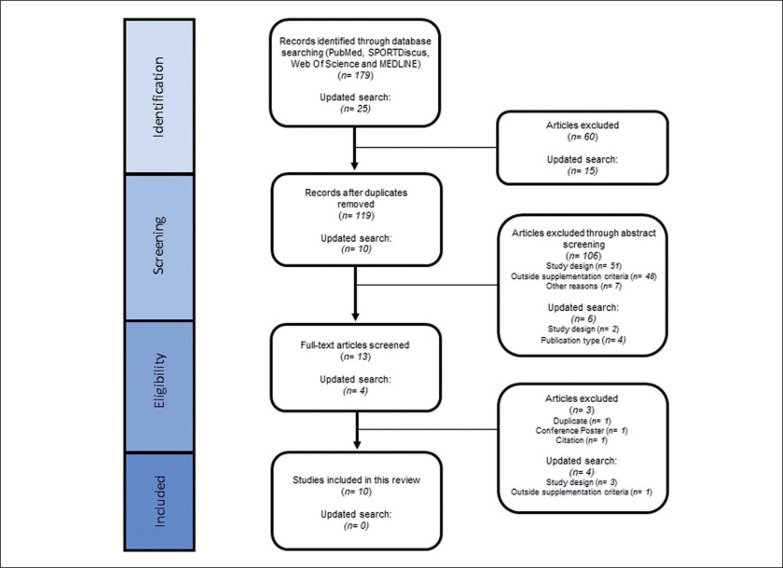
Flowchart of the search strategy and study selection.

The initial search identified 179 articles and after duplicates were removed reduced to 119 (Figure 2). Following title and abstract screening, this number was reduced to 13 which were screened in their entirety. A total of 10 articles of these 13 articles were included in this review after full text screening. The later update identified 25 additional studies which was reduced to 10 after removal of duplicates, and down to 4 for full-text screening. No additional studies were included into this review after the second screening.

### Descriptives

The full analysis included 10 studies [25; 37-41; 50-53] with 12 out-comes for each supplement intervention in total, comprehending 243 individuals. Only 1 out of the 10 studies included females. Participant age ranged from 18–45 years between studies. Studies were performed across four different regions: United Kingdom (3), Australia (3), Brazil (3) and Finland (1). Exercise modalities comprised cycling (5 outcomes), rowing (1 outcome), swimming (4 outcomes) and intermittent running (2 outcomes). Eight of the outcomes were obtained from trained participants and 4 from untrained. In six of the outcomes, beta-alanine was supplemented for ≤ 4 weeks and > 4 in the remainder. Most of the exercise tests employed (n = 11) had a duration between 30 s to 10 minutes, and only 1 had a duration < 30 s, meaning most had a strong anaerobic component [1].

### Meta-analysis

**FIG. 3:**
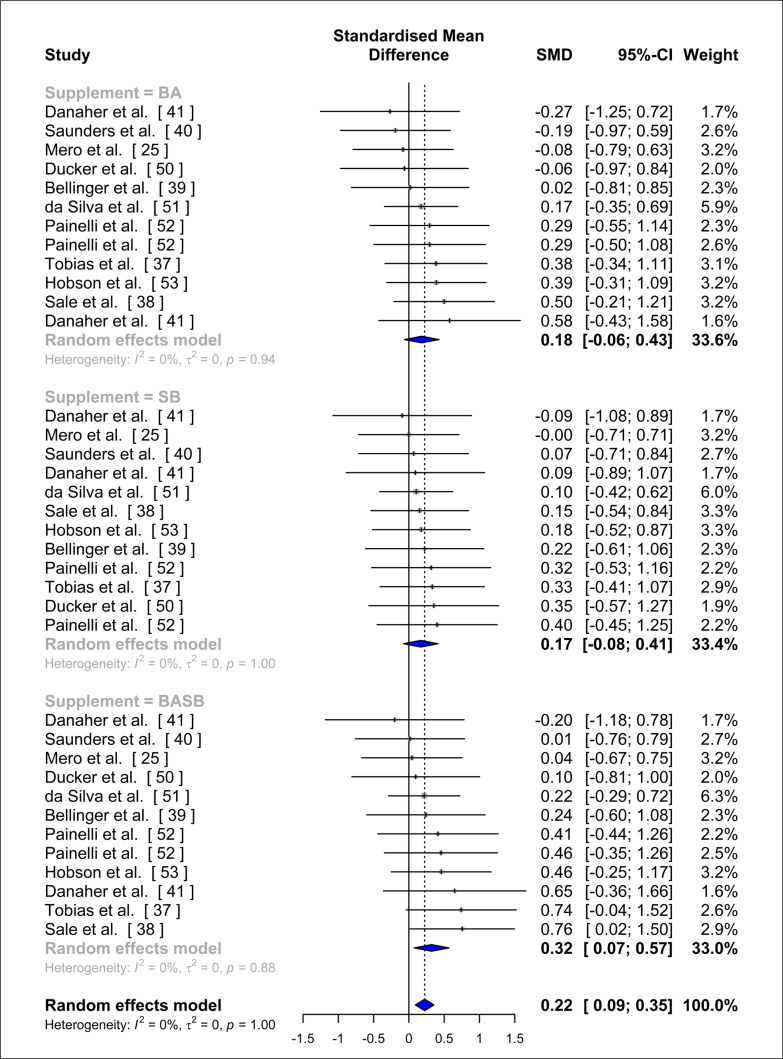
Forest plot of the effect of beta-alanine (BA) alone, sodium bicarbonate (SB) alone, and beta-alanine and sodium bicarbonate (BASB) in combination.

**FIG. 4:**
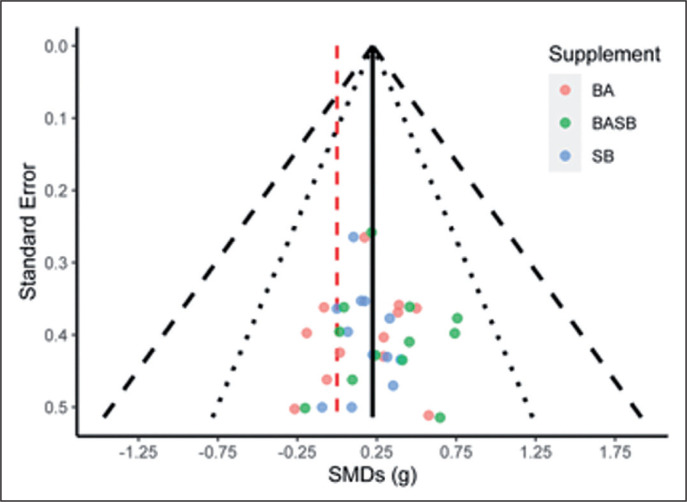
Funnel plot representing effect sizes and their variances according to supplement intervention. The solid black line represents the mean SMD; the dashed red line indicates an SMD of zero. The inner and outer dashed lines represent 95% and 99% confidence intervals. SMDs = Standardised mean ifferences, BA = Beta-alanine, SB = Sodium bicarbonate, BASB = Beta-alanine and sodium bicarbonate.

A forest plot summarizing the results of this study is represented in Figure 3. No effect was detected in this meta-analysis for BA (SMD = 0.18, 95% CI: -0.06; 0.43, p = 0.13, tau^2^ = 0, tau = 0, I^2^ = 0.0%) or SB (SMD = 0.17, 95% CI: -0.08; 0.41, p = 0.16, tau^2^ = 0, tau = 0, I^2^ = 0.0%) separately. However, an effect was seen for the combination of both SB and BA (SMD = 0.32, 95% CI: 0.07; 0.57, p = 0.02, tau^2^ = 0, tau = 0, I^2^ = 0.0%). A meta-regression did not identify any significant differences between supplementing with both BA or SB separately, or the combination of BA and SB (F = 0.58; p = 0.57). No asymmetry could be detected on the funnel plot derived from this meta-analysis, thus, publication and small-study bias were discarded (Figure 4).

## DISCUSSION

The results of this meta-analysis showed that supplementing with beta-alanine and sodium bicarbonate together lead to benefits in exercise performance compared to a placebo, while there was no benefit in taking each of these supplements individually. These meta-analytic data confirm previous findings on co-supplementation [14], but contradict others regarding their individual efficacy [10; 12; 14].

Beta-alanine and sodium bicarbonate are considered effective ergogenic aids and are considered one of only five supplements that are worthwhile for performance as per the International Olympic Committee’s position stand [54]. Furthermore, numerous meta-analyses have shown that both beta-alanine [13; 14] and sodium bicarbonate [10; 12; 18] can improve exercise performance. These meta-analyses were built upon a substantial literature demonstrating the performance-enhancing capacity of beta-alanine and sodium bicarbonate for rowing [20; 21; 22; 23], swimming [16; 24; 25], cycling [26; 27; 28; 29], running [30; 31], team sports [32; 33] and combat sports [34; 35; 36]. Thus, these current results appear in direct contrast to previous work, but may be explained by the low number of studies and outcomes included here. The previous largest beta-alanine meta-analysis was based upon 70 outcomes from 40 studies [14], while the most recent sodium bicarbonate metaanalysis was based upon 192 outcomes from 158 studies [10]. Twelve outcomes from ten studies were included in the current analysis; since only small effects are expected from nutritional supplements, the current analysis was likely underpowered. Indeed, the mean effect statistic for beta-alanine (SMD = 0.18) and sodium bicarbonate (SMD = 0.17) are very similar to previous positive meta-analyses (SMD = 0.18 for beta-alanine, [14]; SMD = 0.19 for sodium bicarbonate, [10]), although confidence intervals were wider from imprecision due to low sample sizes and study numbers. Thus, the lack of a significant effect of each supplement in isolation is likely due, in part, to the low number of studies included and low samples sizes within each study.

The combination of beta-alanine and sodium bicarbonate did lead to a small overall benefit on exercise outcomes. These data confirm a previous meta-analysis that showed that the addition of sodium bicarbonate to beta-alanine provided greater improvements than beta-alanine alone [14]. Several experimental studies also showed improved performance compared to baseline or a placebo when co-supplementing beta-alanine and sodium bicarbonate. Painelli et al. [24] showed a +2.13% performance improvement in 200 m swimming, Bellinger et al. [39] showed a +3.3% improvement in 4 min cycling performance, while both Sale et al. [38] and Danaher et al. [41] showed improvements of +16% in exercise capacity during a high-intensity cycling capacity test performed to exhaustion. Several individual studies did not report statistically significant additive effects of combined combination (i.e., compared to beta-alanine or sodium bicarbonate alone), though this might be due to studies with low samples sizes which were not sufficiently powered to detect small additive differences, if any exist. For example, Sale et al. [38] did not show any significant differences between combined supplementation (+16.2%) and beta-alanine (+12.1%) or sodium bicarbonate (+6.5%) alone. By pooling these data here via meta-analysis, it has been shown that the combination of these two supplements may be worthwhile for athletic populations since this generated the greatest improvements. This is supported by studies reporting further benefits of co-supplementation on repeated upper-body Wingate [37] and 2000 m rowing [20] performance. This is good news for athletes since data suggest they frequently engage in concurrent use of several supplements [55; 56]. The current data suggest that if these two products are beta-alanine and sodium bi-carbonate, performance benefits are likely to occur.

In this meta-analysis we did not include analysis of changes in muscle carnosine content. Only one included study measured changes in muscle carnosine [41]. Although there may be some individual variation in the extent of these changes [57], all studies used recommended dosing strategies of between 3.2 and 6.4 g · day^−1^ and meta-analytical data suggest that all individuals increase muscle carnosine content with chronic beta-alanine supplementation [8] and that these doses lead to improved exercise performance [14]. Thus, this is unlikely to have substantially influenced the results. The current study did not determine the changes in blood bicarbonate concentration following sodium bicarbonate supplementation. Nine studies employed an acute dose as per recommendations (0.3 g · kg^−1^ BM) between 1 and 3 h prior to exercise, which will likely have sufficiently increased blood buffering capacity to elicit performance improvements [58]. Only one study used chronic dosing for sodium bicarbonate [37]. Meta-analytic data suggest that acute and chronic supplementation may be equally effective for exercise performance [10], nonetheless, it is possible that using a supplementation strategy in which exercise was initiated following attainment of peak blood bicarbonate values, performance improvements may have been optimised [59].

### Practical implications

The combination of beta-alanine and sodium bicarbonate was shown to be an effective strategy to improve high-intensity exercise performance, and competitive athletes may wish to engage in supplementation to enhance performance during training and competition. Based upon the current data and current recommendations [10; 14; 54], athletes should ingest beta-alanine chronically, taking 6.4 g per day, for a minimum of 4 weeks before expecting performance improvements. To avoid uncomfortable side-effects, this should preferably be staggered over 4 individual 1.6 g doses separated by 3–4 h [60]. Sodium bicarbonate should be ingested at a dose of 0.3 g · kg^−1^ BM between 1 and 3 h prior to initiating exercise [58]. If possible, supplementation should occur in gastroresistant capsules and alongside a carbohydrate-rich meal to minimise any potential side-effects associated with sodium bicarbonate [61; 62].

## CONCLUSIONS

The results of this meta-analysis showed that supplementing with beta-alanine and sodium bicarbonate together leads to benefits in exercise performance compared to a placebo, although there was no apparent benefit in taking each of these supplements individually.

## Conflict of interest

André Guedes da Silva (2019/22249-7), Gabriel Barreto (2020/ 12036-3) and Bryan Saunders (2021/06836-0) acknowledge the receipt of personal research grants from São Paulo Research Foundation (FAPESP). Bryan Saunders has also previously received financial support from Natural Alternatives International (NAI), a company that produces beta-alanine, to undertake research. NAI has also provided supplements free of charge for experimental investigations and supported open access page charges for numerous publications involving Bryan Saunders. Bryan Saunders has also received remuneration for blog posts and GRAS relating to beta-alanine supplementation and carnosine, and financial support to attend the International Congress on Carnosine and Anserine 2017 in Kentucky, USA. Bryan Saunders has also received sodium bicarbonate supplements free of charge from Umara^®^ (Sweden) and Farmácia Analítica (Brazil) for experimental investigations. These companies have not had any input (financial, intellectual, or otherwise) into the current article. The other authors declare that they have no conflicts of interest relevant to the content of this review.
